# Reduced Sphingosine in Cystic Fibrosis Increases Susceptibility to *Mycobacterium abscessus* Infections

**DOI:** 10.3390/ijms241814004

**Published:** 2023-09-12

**Authors:** Fabian Schnitker, Yongjie Liu, Simone Keitsch, Matthias Soddemann, Hedda Luise Verhasselt, Jan Kehrmann, Heike Grassmé, Markus Kamler, Erich Gulbins, Yuqing Wu

**Affiliations:** 1 Department of Molecular Biology, Institute of Molecular Biology, University Hospital Essen, University of Duisburg-Essen, 45122 Essen, Germany; fabian.schnitker@stud.uni-due.de (F.S.); yong-jie.liu@uk-essen.de (Y.L.); simone.keitsch@uk-essen.de (S.K.); matthias.soddemann@uk-essen.de (M.S.); heike.gulbins@uk-essen.de (H.G.); erich.gulbins@uk-essen.de (E.G.); 2 West German Heart and Vascular Center, Thoracic Transplantation, Department of Thoracic and Cardiovascular Surgery, University Hospital Essen, University Duisburg-Essen, 45122 Essen, Germany; markus.kamler@uk-essen.de; 3 Institute of Medical Microbiology, University Hospital Essen, University of Duisburg-Essen, 45122 Essen, Germany; hedda-luise.verhasselt@uk-essen.de (H.L.V.); jan.kehrmann@uk-essen.de (J.K.); 4 Department of Surgery, University of Cincinnati College of Medicine, Cincinnati, OH 45229, USA

**Keywords:** *Mycobacterium abscessus*, cystic fibrosis, sphingosine, sphingolipids

## Abstract

Cystic fibrosis (CF) is an autosomal recessive disorder caused by the deficiency of the cystic fibrosis transmembrane conductance regulator (CFTR) and often leads to pulmonary infections caused by various pathogens, including *Staphylococcus aureus*, *Pseudomonas aeruginosa*, and nontuberculous mycobacteria, particularly *Mycobacterium abscessus*. Unfortunately, *M. abscessus* infections are increasing in prevalence and are associated with the rapid deterioration of CF patients. The treatment options for *M. abscessus* infections are limited, requiring the urgent need to comprehend infectious pathogenesis and develop new therapeutic interventions targeting affected CF patients. Here, we show that the deficiency of CFTR reduces sphingosine levels in bronchial and alveolar epithelial cells and macrophages from CF mice and humans. Decreased sphingosine contributes to the susceptibility of CF tissues to *M. abscessus* infection, resulting in a higher incidence of infections in CF mice. Notably, treatment of *M. abscessus* with sphingosine demonstrated potent bactericidal activity against the pathogen. Most importantly, restoration of sphingosine levels in CF cells, whether human or mouse, and in the lungs of CF mice, provided protection against *M. abscessus* infections. Our findings demonstrate that pulmonary sphingosine levels are important in controlling *M. abscessus* infection. These results offer a promising therapeutic avenue for CF patients with pulmonary *M. abscessus* infections.

## 1. Introduction

Affecting 1 in 2500 children born in the European Union and the United States, cystic fibrosis (CF) is the most common autosomal recessive disease in Western countries. The disease is caused by mutations in the cystic fibrosis transmembrane conductance regulator gene and the absence of functional protein (human: CFTR, murine: Cftr) [1]. CFTR, a member of the ATP-binding cassette transporter family [2], encodes a phosphorylation-activated anion channel. This channel facilitates the transport of chloride and bicarbonate ions across the apical plasma membrane of epithelial cells. This process, in turn, regulates the movement of water and other ions, such as sodium (Na^+^), across these membranes [3,4,5].

Genetic mutations in *CFTR* lead to various respiratory, reproductive, and gastrointestinal complications. However, the leading cause of morbidity and mortality in these patients is pulmonary destruction upon chronic colonization and infection of the lungs with bacterial pathogens [1].

Recently, nontuberculous mycobacteria have emerged as important pathogens of CF lung disease worldwide [6,7,8]. The incidence of nontuberculous mycobacteria infections in CF patients has increased from 3.3% to 22.6% over the past two decades, resulting in increased morbidity and mortality associated with these pathogens [6,9,10]. However, the incidence of nontuberculous mycobacteria is likely underestimated due to frequent misdiagnosis of nontuberculous mycobacteria infections in developing countries and the lack of data from several countries [11,12].

*Mycobacterium abscessus* ssp. *abscessus* (*M. abscessus* ssp. *abscessus*) is the most common and clinically significant nontuberculous mycobacteria subtype in hospitals and CF centers [6,13,14]. *M. abscessus* ssp. *abscessus* causes respiratory infections (up to 80%), with the highest incidence in CF and immunocompromised patients [6,13]. In addition, *M. abscessus* ssp. *abscessus* is more frequently found in younger CF patients (including children) and patients with severe lung disease than in older and less affected patients [6,13]. Failure to eradicate *M. abscessus* ssp. *abscessus* often leads to accelerated deterioration of lung function.

Inhalation therapy is considered a highly favorable approach for the treatment of airway bacterial infections in CF due to its ability to deliver high doses directly to the target site while minimizing systemic side effects [15]. However, determining the appropriate treatment strategy can be challenging, primarily due to the difficulty in obtaining individual samples to identify the presence of infection accurately [15]. Consequently, exploring a potent bactericidal molecule with broad efficacy could offer a promising option for effectively treating infected individuals.

Lipid imbalances or dyslipidemia have been documented in both individuals with CF and CF mouse models, encompassing a range of lipid species such as fatty acids (e.g. linoleic acid and arachidonic acid) [16,17,18,19], cholesterol [20,21,22,23], and sphingolipids [24,25,26,27]. Among these, the perturbation of sphingolipids, particularly ceramides, has gained significant attention. Studies have indicated an elevation in ceramide levels within the lung epithelia of CF patients and in *CFTR*-mutated epithelial cell lines [26,27]. Analyses of whole lung homogenates from CFTR knockout mouse models exhibited an increased ratio of long-chain ceramides to very long-chain ceramides [25]. The disruption in ceramide levels has been linked to the promotion of pro-inflammatory responses and heightened susceptibility to bacterial infections in individuals with CF [24].

Sphingosine, a lipid derived from ceramide, has been previously shown to exhibit antibacterial properties against several bacterial species, including *P. aeruginosa*, *Escherichia coli*, *Haemophilus influenzae*, *Moraxella catarrhalis*, *Burkholderia cepacia*, *S. aureus*, *Acinetobacter baumannii*, and *Porphyromonas gingivalis* [24,28,29,30,31,32]. In addition, we have also demonstrated an important role of sphingosine in the immediate defense against bacterial infections in the respiratory tract [28,29]. Recent studies, including our own, have found significant changes in the lipid composition of airway epithelial cells in CF, particularly an increase in ceramide and a decrease in sphingosine in the luminal membrane of tracheal and bronchial epithelial cells [24,27,28,29,30,33,34,35,36,37]. Specifically, the deficiency of sphingosine in airway epithelial cells of CF patients or mice with CF has been linked to increased susceptibility to infections caused by *Staphylococcus aureus* (*S. aureus*) and *Pseudomonas aeruginosa* (*P. aeruginosa*) infections [28,29,30].

Given the unique composition of mycobacteria, which includes a thick envelop characterized by very long chain fatty acids, glycolipids, lipoglycans, sulfoglycolipids, mannosides, lipomannan, lipoarabinomannan, lipoproteins, neutral polysaccharides, and mannan, mycobacteria are known to be particularly resistant to many antibiotics [38]. Consequently, we set out to investigate whether sphingosine also affects nontuberculous mycobacteria in vitro and in vivo.

Here, we found that both macrophages and human or mouse epithelial cells lacking CFTR/Cftr eliminate *M. abscessus* ssp. *abscessus* less efficiently than the corresponding wildtype cells. Furthermore, we infected wildtype and *Cftr*-deficient mice with *M. abscessus* ssp. *abscessus* and observed that *Cftr*-deficient mice are highly susceptible to *M. abscessus* ssp. *abscessus* infections. We demonstrate that the increased infection susceptibility of CF cells and mice to *M. abscessus* ssp. *abscessus* correlates with the reduction of sphingosine in CF cells. In vitro treatment of *M. abscessus* ssp. *abscessus* with sphingosine leads to bacterial death. The restoration of sphingosine in human and mouse tracheal or bronchial epithelial cells or macrophages restored the ability of these cells to kill the bacteria to levels comparable with those of healthy or wildtype cells. Finally, we show that inhalation of CF mice with sphingosine restored their resistance to *M. abscessus* ssp. *abscessus* infection, providing evidence of the importance of sphingosine in the in vivo killing of *M. abscessus* ssp. *abscessus* in the lungs.

## 2. Results

### 2.1. CF Epithelial Cells and Macrophages Are Susceptible to M. abscessus ssp. abscessus Infection

**Figure 1:**
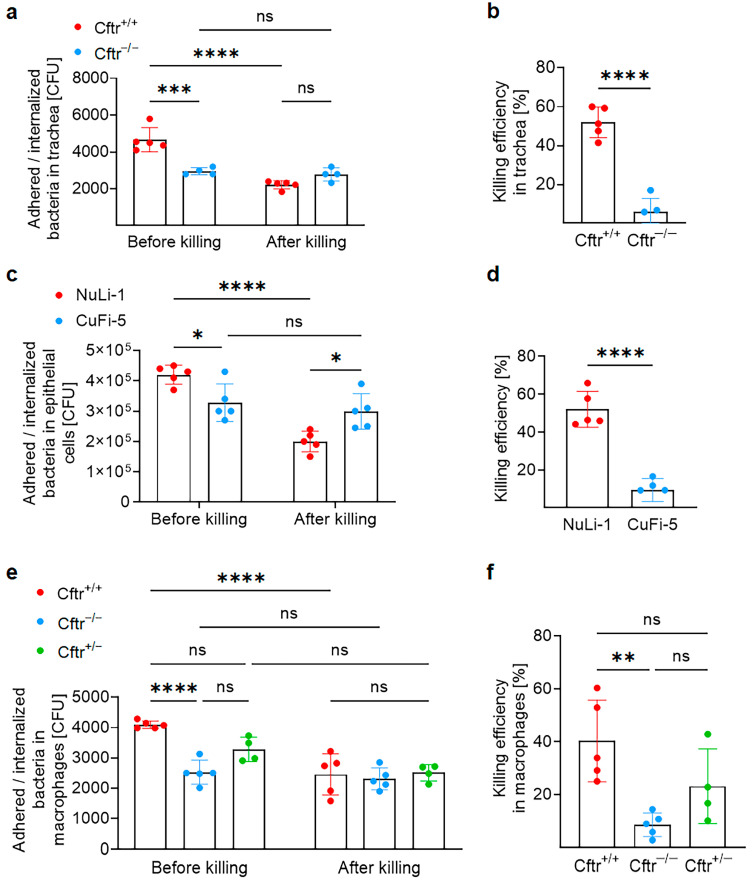
Cftr−deficient epithelial cells and macrophages are more susceptible to *M. abscessus* ssp. *abscessus* infection. (**a**,**b**) Tracheae from wildtype and *Cftr*—deficient mice were isolated and opened, and the epithelial surface was infected with 10^4^ CFU of *M. abscessus* ssp. *abscessus*. After 2 h infection, the tracheae were washed, and the remaining bacteria were quantified through colony—forming unit assay (before killing). To allow killing of the bacteria, the infected and washed tracheae were further incubated in medium for 2 h and bacteria were determined through colony—forming unit assay (after killing). (**b**) The bacterial killing efficiency was calculated as described in the methods. (**c**,**d**) NuLi-1 and CuFi-5 cells were infected with 10^6^ bacteria (MOI 10) for 2 h, washed, and CFU were determined (before killing). Infected and washed cells were further incubated in fresh medium for 2 h, and the bacterial number was determined as after killing. (**d**) The bacterial killing efficiency was then calculated. (**e**,**f**) Macrophages were derived from the bone marrow of wildtype, *Cftr^+/−^* or *Cftr^−/−^* mice and infected with 10^4^ *M. abscessus* ssp. *abscessus*. After 2 h of infection, the cells were washed and the bacteria were quantified (before killing). The washed and infected cells were further incubated for 1 h. The bacterial number was then quantified as shown after killing (**e**). The killing efficiency of bacteria was then calculated (**f**). Shown are the mean ± SD of 4–5 (**a**,**b**), 5 (**c**,**d**), 4–5 (**e**,**f**) independent experiments; * *p* < 0.05, ** *p* < 0.01, *** *p* < 0.001, **** *p* < 0.0001, ns, not significant, *t*-test.

Epithelial cells serve as the first line of defense against pulmonary infection [39]. To investigate the role of Cftr in the defense against *M. abscessus* ssp. *abscessus* infection, we first examined the response of tracheal surface epithelial cells from wildtype (*Cftr^+/+^*) and *Cftr*-deficient mice (*Cftr^−/−^*). To accomplish this, the trachea was isolated and the surface epithelial cells were infected with *M. abscessus* ssp. *abscessus* for 2 h. To remove extracellular bacteria, tracheae were extensively washed 2 h after infection and the adhered plus internalized bacteria were quantified immediately after washing (shown in Figure 1a as “before killing”). Subsequently, the infected and washed tracheae were incubated for an additional 2 h to assess the ability of the cells to eliminate the internalized or adhered bacteria (shown in Figure 1a as “after killing”).

The results indicate that less bacteria adhered to or were internalized by CF epithelial cells during the initial 2 h of infection (Figure 1a before killing). CF epithelial cells then failed to reduce the internalized or adhered bacteria whereas the wildtype tracheal epithelial cells rapidly cleared bacteria (Figure 1a after killing and Figure 1b). These results demonstrate that CF epithelial cells exhibited reduced bacterial killing and allowed for the persistence of bacteria (Figure 1b).

To gain further insights into the role of CFTR in epithelial cells, we conducted experiments involving human epithelial cells NuLi-1 (*CFTR^+/+^*) and CuFi-5 (*CFTR^ΔF508/ΔF508^*). These cells were infected with *M. abscessus* ssp. *abscessus*. Initially, the cells were infected for 2 h and the extracellular bacteria were washed away. Our observation revealed slight variations in the number of the adhered and internalized bacteria between the two cell types (Figure 1c). We then left the washed cells for another 2 h incubation period to assess the killing of the bacteria by these cells. Again, CF cells (CuFi-5 cells) demonstrated a reduced ability to kill the bacteria compared with NuLi-1 cells (Figure 1c,d).

In addition to epithelial cells, macrophages also play a crucial role against bacterial infections during the early or acute stages. To investigate this further, we isolated macrophages from *Cftr*-deficient (*Cftr^−/−^*), *Cftr*-heterozygous (*Cftr^+/−^*), and syngeneic wildtype (*Cftr^+/+^*) mice. Subsequently, these macrophages were infected with *M. abscessus* ssp. *abscessus* for 2 h, followed by extensive washing. Consistent with our previous findings, we observed a higher bacterial count in the wild type macrophages compared with the *Cftr*-deficient macrophages (Figure 1e). Bacterial killing was decreased in *Cftr*-heterozygous and *Cftr*-deficient macrophages compared with wildtype macrophages. In wildtype cells, 40.3% ± 15.5% of *M. abscessus* ssp. *abscessus* were killed within one hour, while *Cftr*-heterozygous killed 23.1% ± 14.2% and *Cftr*-deficient macrophages only killed 8.5% ± 4.5% of the bacteria (Figure 1f). These findings highlight the critical role of Cftr in effective bacterial clearance.

### 2.2. Sphingosine Level Is Reduced in Cftr-Deficient Cells

Previous studies have revealed that sphingosine is essential for eliminating *P. aeruginosa* and *S. aureus* in CF mice [24,28,29,30,31,32]. However, its effects on mycobacteria are presently unknown. Considering our observations that wildtype epithelial cells and macrophages effectively eliminated bacteria, while *Cftr*-deficient cells failed to clear *M. abscessus* ssp. *abscessus*, we hypothesized that sphingosine may play a role in this process.

**Figure 2:**
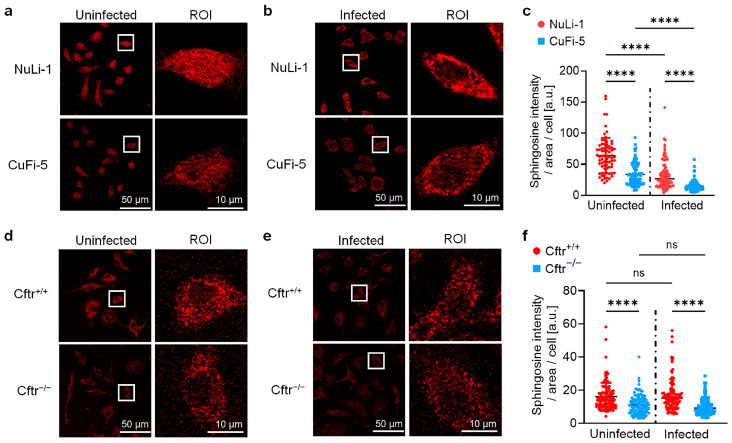
Sphingosine level is reduced in *Cftr*-deficient cells. Representative images of sphingosine staining and the intensity quantification by ImageJ Version 1.53t. (**a**–**c**) NuLi-1 and CuFi-5 cells were either left uninfected (**a**) or infected with *M. abscessus* ssp. *abscessus* for 2 h, washed and further incubated for 2 h (**b**). (**d**–**f**) Bone-marrow-derived macrophages were isolated from *Cftr^+/+^* or *Cftr*^−/−^mice, either left uninfected (**d**) or infected with *M. abscessus* ssp. *abscessus* for 2 h, washed and further incubated for 1 h (**e**). (**c**,**f**) Staining intensity in cells was determined by 10–20 cells per image. Three images from each experiment were quantified. The images were taken using light microscopy and analyzed with ImageJ Version 1.53t. Shown are the mean ± SD of 3 independent experiments; **** *p* < 0.0001, ns, not significant, one-way ANOVA followed by the Tukey test.

To investigate this hypothesis, we performed staining for sphingosine in epithelial cells, specifically NuLi-1 and CuFi-5 cells. We found that CuFi-5 cells, which carry the *Cftr* mutation, exhibited reduced sphingosine levels compared with NuLi-1 cells (Figure 2a,b). Following infection with *M. abscessus* ssp. *abscessus*, CuFi-5 cells maintained lower levels of sphingosine compared with *Cftr* wildtype NuLi-1 cells (Figure 2c,d).

Similarly, we conducted sphingosine staining in *Cftr*-deficient (*Cftr^−/−^*), *Cftr*-heterozygous (*Cftr^+/−^*), and syngeneic wildtype (*Cftr^+/+^*) macrophages. Prior to infection, *Cftr*-deficient macrophages displayed lower sphingosine levels compared with wildtype macrophages (Figure 2e,f). The same disparity was observed after infection with *M. abscessus* ssp. *abscessus* (Figure 2g,h).

Significantly, a noteworthy reduction in sphingosine staining on the cell surface was evident in epithelial cells after infection, which was less pronounced in macrophages. Furthermore, both epithelial cells and macrophages exhibited a repositioning of sphingosine to the cellular periphery, suggesting sphingosine could potentially play a role in the bacterial binding/internalization and elimination process as a bactericidal lipid during *M. abscessus* ssp. *abscessus* infection.

### 2.3. Sphingosine Kills Mycobacterium abscessus ssp. abscessus

**Figure 3:**
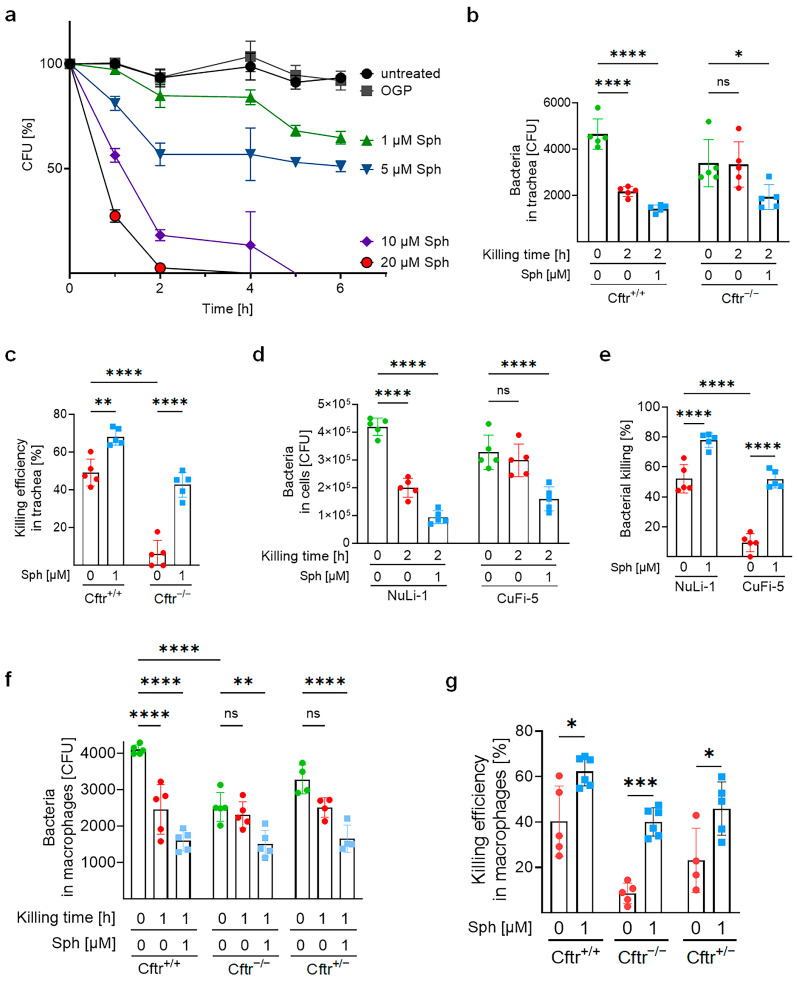
Sphingosine kills *M. abscessus* ssp. *abscessus* in vitro and intracellularly. (**a**) *M. abscessus* ssp. *abscessus* were resuspended in HEPES-buffered RPMI-1640 at a concentration of 10^6^/mL with OGP (octyl-glucopyranoside, solvent of sphingosine) or sphingosine in different concentrations for the indicated time. The bacterial number was quantified from 5–9 independent experiments. (**b**,**c**) Tracheae isolated from wildtype or *Cftr*-deficient mice, (**d**,**e**) NuLi-1 and CuFi-5 cells or (**f**,**g**) bone-marrow-derived macrophages isolated from wildtype, *Cftr^+/−^*, or *Cftr^−/−^* were infected with *M. abscessus* ssp. *abscessus* for 2 h, washed, and CFU were determined and shown as killing for 0 h. The washed and infected cells were then incubated with fresh medium with or without 1 μM sphingosine for 2 h (epithelial cells) or 1 h (macrophages). The killing of bacteria was determined through a colony-forming unit assay. The panels (**b**,**d**,**e**) show the absolute CFU count, and the panels (**c**,**e**,**g**) show the killing efficiency calculated as described in the Section 4. Shown are the mean ± SD of 4–6 independent experiments, * *p* < 0.05, ** *p* < 0.01, *** *p* < 0.001, **** *p* < 0.0001, ns, not significant, 2-way ANOVA followed by the Tukey test.

These data suggest that airway sphingosine might be important in the defense against respiratory tract infections with *M. abscessus* ssp. *abscessus*. To examine this hypothesis, we first treated *M. abscessus* ssp. *abscessus* with sphingosine directly. The results indicate that sphingosine is capable of killing *M. abscessus* ssp. *abscessus* in a dose- and time-dependent fashion (Figure 3a).

Next, we infected mouse tracheal epithelial cells with *M. abscessus* ssp. *abscessus* and added sphingosine or its solvent octyl-glucopyranosid (OGP), respectively, 120 min after initiation of the infection. Samples were then incubated for another 2 h (killing time). Our results show that wildtype cells reduced the number of bacteria within these 2 h by more than 50% (Figure 3b), while *Cftr*-deficient cells were unable to kill the bacteria (Figure 3b). Sphingosine treatment promoted the killing of the pathogen by wildtype tracheal epithelial cells and restored killing of *M. abscessus* ssp. *abscessus* by *Cftr*-deficient tracheal epithelial cells, resulting in a 5-fold increase in killing efficiency compared with untreated CF cells (Figure 3c).

To investigate the effects of sphingosine on human airway epithelial cells, we infected *CFTR*-expressing NuLi-1 and *CFTR*-mutated CuFi-5 cells with *M. abscessus* ssp. *abscessus* and treated them with sphingosine or solvent 2 h after starting the infection. Similar to the tracheal epithelial cells, CuFi-5 cells were unable to kill *M. abscessus* ssp. *abscessus,* while treatment of CuFi-5 cells with sphingosine increased the killing of *M. abscessus* ssp. *abscessus* approximately 5-fold (Figure 3d). Sphingosine even increased the killing of *M. abscessus* ssp. *abscessus* in *CFTR*-wildtype NuLi-1 cells by approximately 30% (Figure 3e).

To prove the role of sphingosine in macrophages, we infected bone-marrow-derived macrophages isolated from wildtype, *Cftr*-deficient, and *Cftr*-heterozygous mice with *M. abscessus* ssp. *abscessus* and left them untreated or we treated the cells with sphingosine for 60 min, starting 120 min after the infection. Our results again indicated that the killing of the bacteria was reduced or almost absent in *Cftr*-deficient cells (Figure 3f), and this killing of *M. abscessus* ssp. *abscessus* in *Cftr*-deficient macrophages was restored by sphingosine (Figure 3f,g).

### 2.4. Sphingosine Levels Are Downregulated in the Lung of CF Mice, Which Are More Susceptible to Pulmonary M. abscessus ssp. abscessus Infection

**Figure 4:**
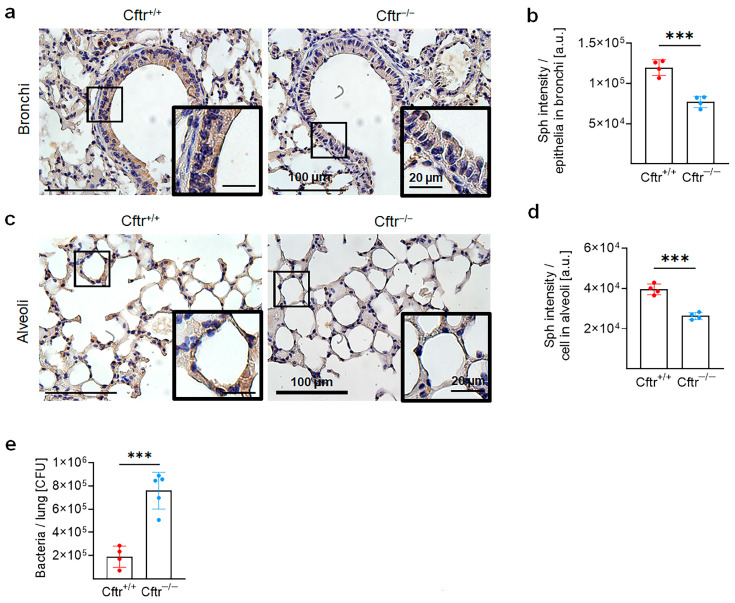
*Cftr*-deficient mice are susceptible to *M. abscessus* ssp. *abscessus* infection and are associated with downregulated sphingosine levels in the lungs. (**a**–**d**) The lungs of wildtype (*Cftr^+/+^*) or *Cftr*-deficient (*Cftr^−/−^*) mice were harvested, embedded in paraffin, and subjected to immunohistochemistry using anti-sphingosine antibodies. Panels (**b**,**d**) represent the quantification of staining in the bronchi (**b**) and alveoli (**d**). Staining intensity in the bronchi was quantified in the entire epithelial layer. Staining intensity in the alveoli was determined in five alveoli per image. From each mouse, 4–7 images were quantified and the average images were taken, *n* = 4. The images were taken using light microscopy and analyzed with ImageJ Version 1.53t. Brown staining indicates positive staining. Shown are mean ± SD, *** *p* < 0.001, *t*-test. Representative images from 4 mice are shown. (**e**) Wildtype or Cftr-deficient mice were intranasally infected with 10^6^ CFU *M. abscessus* ssp. *abscessus* for 6 h and then sacrificed. The lungs were isolated and homogenized and the bacterial load was determined through colony-forming unit assays. Shown are the mean ± SD from *n* = 4–5 independent experiments. Statistical significance was determined through the *t*-test, *** *p* < 0.001.

Next, to gain insights into the mechanisms of pulmonary infection of CF patients with *M. abscessus* and investigate the role of sphingosine and Cftr in this infection, we utilized *Cftr*-deficient mice (*Cftr^−/−^*) as a model. Previous studies have demonstrated that *Cftr^−/−^* mice accumulate ceramide and exhibit reduced sphingosine levels in the bronchi and trachea when compared with wildtype mice (*Cftr^+/+^*), similar to findings in humans [28,29,30]. Here, we confirmed these findings by showing a decrease in sphingosine levels in bronchia and alveoli of *Cftr^−/−^* mice (Figure 4a–d). The reduction of bronchial sphingosine levels in CF mice correlated with a higher number of *M. abscessus* ssp. *abscessus* in the lungs of *Cftr*-deficient mice upon infection compared with infected wildtype mice (Figure 4e).

### 2.5. Inhalation of Sphingosine Reduces Pulmonary M. abscessus ssp. abscessus Infections of CF-Deficient Mice In Vivo

To investigate whether reconstitution of sphingosine in CF mice restores resistance to infection with *M. abscessus* ssp. *abscessus,* we infected CF mice with *M. abscessus* ssp. *abscessus*. After infection, we administered sphingosine via inhalation at a concentration of 10 μM 60 min post infection and evaluated the lung infection status after a 6 h period.

**Figure 5:**
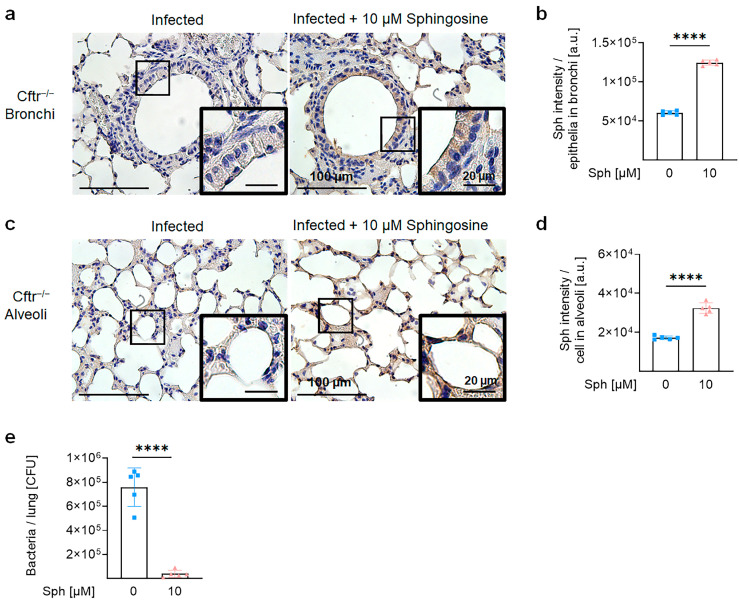
Sphingosine treatment improves killing of *M. abscessus* ssp. *abscessus* in *Cftr*-deficient mice. *Cftr*-deficient mice were infected with *M. abscessus* ssp. *abscessus* (**a**–**d**) for 60 min and then inhaled with either NaCl (square) or sphingosine (triangle). The lungs were isolated 6 h after infection. Panels (**a**,**c**) display representative immunohistochemistry staining of sphingosine from the bronchi (**a**) and alveoli (**c**). Images represent 5 independent experiments and were quantified using Image, J (**b**,**d**). (**e**) Mice were infected with *M. abscessus* ssp. *abscessus* for 60 min and then inhaled with 10 μM sphingosine or left untreated; the lungs were removed after 6 h and homogenized. Bacterial numbers in the lungs were determined through a colony-forming unit assay, *n* = 5, one-way ANOVA, and the Dunnett test. Shown are the mean ± SD of 5 independent experiments; **** *p* < 0.0001, *t*-test.

Sphingosine inhalation led to increased sphingosine levels in both bronchi and alveoli, further confirming the successful delivery of the compound to the target site (Figure 5a–d). The administration of sphingosine resulted in a significant reduction in bacterial counts (CFU) in the lungs. At a concentration of 10 μM sphingosine, the bacterial counts decreased 18.6-fold compared with the untreated but infected CF group (Figure 5e).

### 2.6. Sphingosine Protects Primary Human Tissues and Cells from M. abscessus ssp. abscessus Infection

As *M. abscessus* ssp. *abscessus* is a pathogen that often affects patients with pulmonary diseases, we tested whether treatment with sphingosine reduced infections of human tissues with this pathogen. Thus, we collected human bronchi from lung transplantation surgeries and carefully dissected them into small, uniformly sized pieces. These tissues were either left uninfected or infected with *M. abscessus* ssp. *abscessus* for 1 h. Subsequently, we either left the tissues untreated or treated them with sphingosine for 2 h.

**Figure 6:**
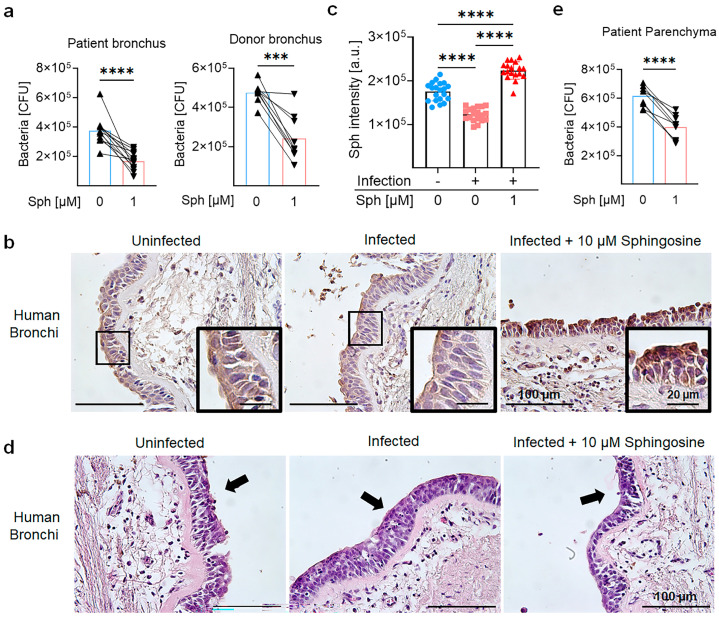
Sphingosine ameliorates *M. abscessus* ssp. *abscessus* infection in human ex vivo bronchi and lungs. (**a**–**d**) Freshly isolated bronchi from donors and patients were either left uninfected (dot) or infected with *M. abscessus* ssp. *abscessus* for 1 h, washed, and then treated with 1 μM sphingosine (triangle) for 2 h or left untreated (square) and also further incubated for 2 h. (**a**) Bronchi were then washed and homogenized, and intracellular bacteria from patients or healthy donor bronchus were quantified through colony-forming unit assay. (**b**) Sphingosine in uninfected or infected donor bronchi was visualized using anti-sphingosine antibodies and immunohistochemistry staining. Representative results from five experiments are shown. (**c**) Staining intensities in 3–4 sections from 5 bronchi were quantified using ImageJ Version 1.53t. (**d**) Uninfected and infected donor bronchi were embedded in paraffin and sectioned, and the sections were stained with H&E. Arrows show the epithelial layer. Shown are representatives from five independent experiments. (**e**) Single cells were prepared from freshly isolated patient lung parenchyma and infected with *M. abscessus* ssp. *abscessus* for 1 h. Cells were then treated with sphingosine for 2 h or left untreated. Finally, the cells were extensively washed and intracellular bacteria were determined through colony-forming assay. The results are from every *n* = 9 sample. Shown are the mean ± SD; *** *p* < 0.001, **** *p* < 0.0001 2-way ANOVA, followed by the Tukey test or, if appropriate, using the *t*-test.

Our results show that *M. abscessus* ssp. *abscessus* infects primary human tissues from both patients and healthy donors ex vivo. Ex vivo treatment of infected bronchi with sphingosine decreased infection in bronchi obtained from 9 patients (recipient, 1 CF, and 8 idiopathic pulmonary fibrosis) and 9 healthy donors (Figure 6a). The controls show that the treatment of bronchi with sphingosine resulted in the incorporation of sphingosine by the bronchial epithelial cells (Figure 6b,c). The epithelial layer remained intact after infection and treatment with sphingosine, indicating the potential of sphingosine to preserve tissue integrity during treatment (Figure 6d).

Furthermore, we collected lung parenchyma from patients’ lungs and prepared single cells. These primary human lung cells were infected with *M. abscessus* ssp. *abscessus* and either left untreated or treated with sphingosine. Similar to the findings in the bronchi, sphingosine treatment significantly augmented the killing of *M. abscessus* ssp. *abscessus* through primary human lung cells, highlighting the bactericidal properties of sphingosine (Figure 6e).

## 3. Discussion

Our study suggests that reduced levels of sphingosine in CFTR/Cftr-deficient airway cells contribute to the high susceptibility to *M. abscessus* ssp. *Abscessus* infection of CF mice and CFTR-deficient human bronchial and lung tissues. Different cells and animal models demonstrate that CFTR/Cftr regulates sphingosine levels in human and murine epithelial cells and macrophages. Sphingosine contributes to the elimination of *M. abscessus* ssp. *Abscessus*, the improvement of lung pathology, and defenses against pathogenic infection.

The present study also confirmed previous studies demonstrating that sphingosine is reduced in cells and tissues lacking CFTR/Cftr [28]. We detected reduced sphingosine levels in the lungs of CF mice and in macrophages isolated from these mice. Sphingosine is metabolized from ceramide by ceramidase. Apart from the lack of sphingosine in CF tissues and cells, previous studies have demonstrated an increase in ceramide in CF epithelial cells [27]. A previous study showed that β1-integrin is trapped in ceramide-enriched membrane domains/ceramide platforms in the luminal membrane of upper-airway epithelial cells from CF mice and patients [29]. The accumulation of β1-integrin in ceramide-enriched membrane platforms downregulates acid ceramidase expression and leads to a reduction in surface sphingosine, which kills bacteria. Normalizing β1-integrin, acid ceramidase, or sphingosine ameliorated CF-associated *P. aeruginosa* infections [29].

Bernut et al. (2016) demonstrated an important role of Cftr and the innate immune system in controlling *M. abscessus* ssp. *abscessus* infections in zebrafish [40]. We confirm the central role of CFTR/Cftr in preventing and controlling *M. abscessus* ssp. *abscessus* infections in mice in vivo and in human freshly isolated lung tissue ex vivo. We also demonstrate that deficiency of Cftr renders macrophages more susceptible to *M. abscessus* ssp. *abscessus* infections. The studies on zebrafish indicated that the loss of Cftr increases the host’s vulnerability to *M. abscessus* ssp. *abscessus* through impaired NADPH oxidase activity and lack of production of reactive oxygen species––mechanisms that restrict bacterial growth in Cftr-positive cells [40]. Here, we indicate that sphingosine also plays an important role in killing *M. abscessus* ssp. *abscessus* through tracheal, bronchial, and alveolar epithelial cells as well as macrophages. Sphingosine has a direct effect on the bacteria [41], but it is possible that sphingosine in vivo may promote other mechanisms that kill the bacteria, for instance, by activating NADPH oxidases. Ceramide has been previously shown to regulate reactive oxygen species in different cellular processes, including infection [42,43]; however, the interaction of sphingosine with NADPH oxidase remains to be determined.

Sphingosine has been shown to exhibit antimicrobial activity against various bacteria [24,28,29,30,31,32,44,45,46]. Our results extend the bactericidal activity of sphingosine to *M. abscessus* ssp. *abscessus*. Sphingosine was shown to kill extracellular bacteria by rapid membrane permeabilization [41]. This is consistent with recent electron microscopy studies investigating the morphology of *P. aeruguinosa* upon treatment with sphingosine [47]. These studies also demonstrated the rapid permeabilization of the bacteria, resulting in their disruption within 1 h of sphingosine treatment.

Interestingly, our results show a reduction of the uptake and/or binding of *M. abscessus* ssp. *abscessus* into CFTR-deficient epithelial cells and macrophages, as evident in Figure 1 before killing. This discovery supports earlier studies where Pier and colleagues demonstrated that epithelial cells with CFTR mutations exhibited impaired uptake of *Pseudomonas aeruginosa* when compared with wild type cells [48,49]. The uptake of bacteria is important for subsequent bacterial eradication, as the initial internalization of bacteria through endocytosis stimulates immune responses and activates NF-κB pathways [50]. Hence, the reduced uptake and binding of *M. abscessus* could be linked to CFTR deficiency, potentially resulting in the failure to trigger an effective immune response and ultimately rendering the host more susceptible to bacterial infection. Moreover, we observed a reduction and translocation of sphingosine, particularly in epithelial cells, toward the cellular periphery following infection, which raises the possibility of sphingosine potentially aiding in the binding and internalization of *M. abscessus*. Sphingosine has the capacity to directly eliminate a range of bacteria and can bind to bacteria via cardiolipin [41], underscoring the direct interaction between sphingosine and bacteria. In the context of infection, it is plausible that sphingosine emerges at the cellular surface to serve as a bridge for bacterial binding. Nonetheless, additional investigations are needed to elucidate the involvement of sphingosine and CFTR in the internalization and endocytosis of *M. abscessus*.

Here, we show that sphingosine also kills or facilitates the killing of *M. abscessus* ssp. *abscessus*. It is unknown how endogenous sphingosine acts on intracellular bacteria. The staining and quantification of sphingosine levels in the cells revealed that sphingosine levels are reduced after infection in epithelial cells but not in macrophages. This indicates that sphingosine may function differently depending on the cell type. In epithelial cells where sphingosine is more abundant, it might be directly consumed in defending against the infection. However, in macrophages, it is possible that sphingosine accumulates in phagosomes, as shown for viral infections [51], and directly kills pathogens in these phagosomes. Alternatively, it may also be possible that sphingosine promotes the fusion of phagosomes with lysosomes to kill intracellular pathogens or sphingosine alters the intracellular metabolism, for instance, to produce reactive oxygen radicals. Furthermore, sphingosine can be phosphorylated to sphingosine-1-phosphate (S1P), which has demonstrated potential in combating mycobacterial infections [52]. During *Mycobacterium tuberculosis* infection, S1P has been shown to stimulate the innate immune response, enhance the expression of iNOS proteins within macrophages, and facilitate the migration of CD11b+ alveolar macrophages to the infected lung [52]. Therefore, the administration of sphingosine to the infected hosts could be phosphorylated into S1P, thereby bolstering the innate immune response.

Our studies provide evidence that sphingosine or its derivatives might serve as a therapeutic agent for CF patients with pulmonary bacterial infections, in particular, with *M. abscessus* ssp. *abscessus*. In this context, it is important to note that we also used human lungs and bronchi to demonstrate the possibility of sphingosine being applied to patients. We observed a marked antibacterial effect of sphingosine. In fact, *M. abscessus* ssp. *abscessus* infection is not limited to CF patients but also occurs in patients with chronic obstructive pulmonary disease or non-cystic fibrosis bronchiectasis, as well as occurring in immunocompromised hosts [8,11]. Therefore, these ex vivo samples from lung transplantations allowed us to assess the safety and usability of sphingosine in human-infected bronchus or lungs.

These findings not only support the notion that sphingosine can act as a potent bactericidal agent but also, for the first time utilizing human transplantation materials, underscore its versatility and potential for broader therapeutic applications. Incorporating sphingosine into inhalation therapy could serve as a valuable addition to the existing arsenal of drugs, providing an alternative treatment option with the potential to combat respiratory infections more effectively. Further research and clinical investigations are warranted to explore the full potential and efficacy of sphingosine as a therapeutic molecule in the field of respiratory medicine.

## 4. Materials and Methods

### 4.1. Human Samples

All studies on human tissues were approved by the Ethical Board of the Medical Faculty, University of Duisburg-Essen, Germany, under the registration number 17-7326-BO.

Bronchial pieces from human lung grafts were freshly removed and immediately transported for preparation. Briefly, the bronchi were cut into similar-sized pieces of 10 × 10 mm, washed in DMEM (Gibco, Paisley, UK), and infected with 3.5 × 10^5^ bacteria, prepared as described below, for 1 h. The samples were extensively washed, fresh DMEM medium containing 1 μM sphingosine or DMEM medium was added, and incubation was continued for 2 h. Samples were either fixed in 4% paraformaldehyde (PFA) for further experiments or lysed by adding saponin to a final concentration of 0.5% at 37 °C for 15 min. The lysed material was plated on LB agar. Colonies were counted after 3 days of incubation at 37 °C.

The lung parenchyma tissues were aseptically transferred to a sterile petri dish and finely minced using a pair of sterile scissors. The minced tissues were then transferred to ice-cold PBS to remove any remaining blood. The tissues were incubated in 0.6 mg/mL collagenase (Merck; # C2-22-BC), 0.02 mg/mL Elastase (Serva; # 20930.01), and 17 units/mL DNase (QIAGEN, # 79254) at 37 °C for 30 min. After the incubation, the samples were washed with PBS and the cell suspension was passed through a 70 μm strainer and centrifuged at 300× *g* for 5 min at 4 °C. The cell pellet was resuspended in red blood cell lysis buffer (Biolegend) for 2 min at room temperature, centrifuged again, and resuspended in RPMI1640 medium for infection.

### 4.2. Mouse Experiments

All the studies were performed in accordance with the NRW State Office for Nature, Environment and Consumer Protection (LANUV), Recklinghausen, Germany, under the number AZ-81.02.04.2019.A148 or the local IACUC, Cincinnati, OH, USA.

In our study, we used *Cftr^tm1Unc^-Tg^(FABPCFTR)^* (abbreviated *Cftr^−/−^*) Jaw mice (Jackson Laboratories, Bar Harbor, ME, USA) that were transgenic for *CFTR* in the intestine under control of the FABP (human fatty acid binding protein 1 liver (FABP1) promoter. We used mice at 24 to 40 weeks of age. Expression of CFTR in the intestine allows normal development and feeding with a normal diet. The *CFTR* knockout assumes the insertion of a neomycin selection cassette into exon 10 at sequences corresponding to codon 489 of the encoded protein.

### 4.3. M. abscessus ssp. abscessus Preparation

*M. abscessus* ssp. *abscessus* (ATCC 19977) was used for all in vivo and in vitro infections. For the experiments, bacteria were shaken at 120 rpm at 37 °C in Erlenmeyer flasks containing 10 mL Middlebrook 7H9 Broth supplemented with glycerol (BD Biosciences, Heidelberg, Germany). After 16 to 24 h incubation, the bacteria were used for infection experiments. The suspended bacteria were collected by centrifugation at 3000× *g* for 10 min, the bacterial pellet was resuspended in PBS buffer and vortexed for 1 min. The bacteria were then bath-sonicated for 5 min at 4 °C to suspend any clumps. Unseparated bacterial aggregates were removed by centrifugation at 220× *g* for 2 min. The supernatant containing single *M. abscessus* was carefully collected. We calculated the bacterial count based on the OD using an Eppendorf BioPhotometer 6131 (OD_600_ 1 = 10^8^ bacteria).

### 4.4. Mouse Infection

*Cftr*-deficient (*Cftr^−/−^*) CF mice and syngenic wildtype (*Cftr^+/+^*) littermates were intranasally infected with 1 × 10^6^ *M. abscessus*. The bacteria were prepared as described above and resuspended in 0.9% NaCl to a concentration of 1 × 10^6^/50 μL. The mice were anesthetized with isoflurane for a short period and infected with 50 μL of the prepared bacterial solution intranasally drop by drop. The mice were sacrificed by cervical dislocation after 6 h, the lungs were removed, and the right upper lobe was homogenized to determine CFU.

The remaining lung tissue was fixed in 4% PFA and embedded in paraffin after serial dehydration with an Ethanol to Xylol gradient.

To investigate the in vivo effect of sphingosine on bacteria, the mice were infected as described above. After 1 h of infection, the mice were inhaled for 10 min with 1 mL 0.9% NaCl or 1 mL 10 μM sphingosine (dissolved in 0.9% NaCl). After 6 h of infection, the mice were sacrificed and samples were collected as above.

### 4.5. Mouse Inhalation

Inhalation was performed using a PARI Boy SX nebulizer (PARI GmbH, Starnberg, Germany), which generates a fine aerosol by pumping the fluid with an air jet. The mice were inhaled with the aerosol via a mask which is part of an oral inhalation device for children (LL-Nebulizer); the mask was clipped at the sides to cover only the nose and the surrounding part of the face. The mice were inhaled with 1 mL of 0.9% NaCl containing 10 μM sphingosine.

### 4.6. Sphingosine Preparation

Sphingosine (Avanti Polar Lipids, #860490P) was resuspended in 7.5% n-octyl-β-D-glucopyranoside (OGP) at 20 mM concentration, sonicated to obtain a suspension and promote the formation of micelles, and stored at −20 °C. Before each experiment, sphingosine was sonicated (Bandelin Sonorex) for 10 min and diluted in 0.9% NaCl to 1 μM or 10 μM sphingosine.

### 4.7. Colony-Forming Unit Assay

To determine CFU (colony-forming unit) in vivo, the right upper lobes of the lungs from infected mice were transferred into a 6-well plate, and 0.5% saponin/well was added. To determine CFU in vitro, 0.5% saponin was added to cells or tracheae. After 15 min of incubation at 37 °C, the lysed material was diluted with PBS and aliquots were plated on LB agar. The colonies were counted after 3 days in the incubator at 37 °C.

### 4.8. Immunohistochemistry Staining

Samples were cut at 6 μm, deparaffinized, rehydrated, and boiled with citrate buffer (BioLegend; #420902) for 10 min. After cooling, the samples were treated with 0.3% hydrogen peroxide for 10 min. Next, they were washed twice with PBS and blocked for 10 min at room temperature with PBS, 0.05% Tween 20 (Sigma, St. Louis, MO, USA), and 5% fetal calf serum (FCS). Samples were stained with anti-sphingosine (1:100 dilution, Cosmobio, Tokyo, Japan; #ALF-274042010) in PBS, 0.05% Tween 20, and 1% FCS at 4 °C overnight. The samples were washed three times with PBS plus 0.05% Tween 20. We secondary-labeled the tissues for 45 min at room temperature with ZytoChem Plus (HRP) One-Step Polymer anti-mouse/rabbit (Zytomed Systems, Berlin, Germany; #ZUC053-100) in PBS, 0.05% Tween 20, and 1% FCS. The tissues were washed, then the DAB substrate (Pierce DAB-Substrate Kit, Thermo Fischer Scientific, Waltham, MA, USA; #34002) was added to the samples for 4 min at room temperature. The samples were counterstained with hematoxylin for 2 min. Finally, the tissues were dehydrated with ethanol to xylene and mounted with Eukitt.

### 4.9. Hematoxylin and Eosin Staining

For hematoxylin and eosin (H&E) staining, samples were sectioned at 6 μm, dewaxed, and rehydrated as described above, and stained for 5 min with Mayer’s hemalaun solution (T865.1; Roth, Karlsruhe, Germany). Samples were rinsed with water for 15 min, stained for an additional 2 min with 1% eosin solution, washed with water, dehydrated with ethanol to xylene, and embedded in Eukitt mounting medium (Sigma-Aldrich, Burlington, MA, USA).

### 4.10. Trachea Infection

To isolate the trachea, the mice were sacrificed and the trachea was surgically removed. Subsequently, the trachea was infected with 1 × 10^4^ *M. abscessus* ssp. *abscessus* for 2 h. Bacteria were applied to the surface of the trachea in RPMI-medium (Gibco, Paisley, UK). The trachea was placed in a humidified chamber and incubated at 37 °C. After 2 h incubation, the tracheae were extensively washed and the remaining bacterial numbers were determined by adding saponin to the wells to reach a final concentration of 0.5%. Samples were incubated at 37 °C for 15 min, and the lysed material was diluted and spread onto LB agar plates to determine the intracellular bacterial number (before killing CFU). To assess the bacterial killing efficiency, cells infected for 2 h and subsequently washed were either left untreated or treated with 1 μM sphingosine for another 2 h. Finally, the tracheae were homogenized, and the CFU were determined as a measure of the after-killing bacterial number. The killing efficiency was calculated using the following formula:

(1) $$Killing\;efficiency=\frac{\left(before\;killing\;CFU-after\;killing\;CFU\right)}{before\;killing\;CFU}$$

### 4.11. Bone-Marrow-Derived Macrophages (BMDM)

To obtain BMDM, mice were sacrificed and femurs and tibias were flushed with minimal essential medium (MEM; Gibco, Paisley, UK), supplemented with 10% FCS (Gibco), 10 mM HEPES (Roth GmbH, Karlsruhe, Germany; pH 7.4), 2 mM L-glutamine, 1 mM sodium pyruvate, 100 μM nonessential amino acids, 100 U/mL penicillin, and 100 μg/mL streptomycin (Gibco). The isolated cells were passed through a 23-G needle to obtain single cells, and 3–6 × 10^6^ cells were cultured in petri dishes in MEM containing 20% L-cell supernatant as a source of macrophage colony-stimulating factor (M-CSF). Fresh MEM/L-cell supernatant medium was applied every 3 days of culture. Macrophages matured within the next 6 days and were used on day 8 of culture. Cells were grown at 37 °C in 5% CO_2_.

### 4.12. NuLi-1 and CuFi-5

NuLi-1 (wildtype CFTR, ATCC CRL-4011) derived from the normal human airway epithelium, and CuFi-5 (ATCC CRL-4016) derived from the bronchial epithelium of a homozygous CFTR F508del/F508del individual were grown on human placental collagen type VI (60 μg/mL) (Sigma, St. Louis, MO, USA) coated flasks in PneumaCult-EX-medium (Stemcell Technologies, Cologne, Germany, #05008) supplemented with 0.48 μg/mL hydrocortisone (Stemcell Technologies #07926) at 37 °C in 5% CO_2_.

### 4.13. Infection

For the BMDM, on the day of infection, the medium was changed to MEM supplemented with 10 mM HEPES (Roth GmbH, Karlsruhe, Germany; pH 7.4) and 5% fetal bovine serum (Gibco). Cells were left uninfected or infected with *M. abscessus* prepared as above. Synchronous infection conditions and enhanced interactions between bacteria and host cells were achieved by centrifuging the bacteria at 55× *g* onto the cells for 5 min. The end of centrifugation was defined as the starting point of infection. To study bacterial killing by macrophages, cells were seeded at 1 × 10^4^ per well and infected at a multiplicity of infection (MOI) ratio of 1:1. To study bacterial killing by NuLi-1 and CuFi-5 cells, they were seeded at 1 × 10^5^ per well and infected at MOI ratio of 1:10.

After 2 h of infection, the cells were washed off using 3 changes of medium, and either proceeded further or the bacterial number was determined. To determine the bacterial number before killing, the cells were lysed by adding 0.5% saponin for 15 min and the lysates were diluted and spread on LB agar plates, or the infected and washed cells were left untreated or treated with 1 μM sphingosine at 37 °C for 1 h for macrophages and 2 h for epithelial cells. The bacterial number after killing was determined in the same way as described above.

### 4.14. Immunofluorescence Staining

NuLi-1, CuFi-5, and bone-marrow-derived macrophages were left uninfected or infected as above. The cells were then washed with PBS, fixed in 4% PFA for 10 min, and washed with PBS. Next, they were blocked for 10 min at room temperature with 5% FCS in PBS. The samples were stained with anti-sphingosine (1:2000 dilution, Cosmobio; #ALF-274042010) in 1% FCS-PBS at room temperature for 1 h. The samples were washed and secondary-labeled for 45 min at room temperature with Cy5-coupled donkey anti-mouse IgM (Jackson ImmunoResearch, Wes Grove, PA, USA, #715-176-020). The cells were washed three times with PBS plus 0.05% Tween 20, then mounted with DAPI—aqueous mounting medium (Abcam, Cambridge, UK, ab104139).

### 4.15. Direct Treatment of M. abscessus ssp. abscessus with Sphingosine

*M. abscessus* ssp. *abscessus* was prepared as described above and the OD was measured. Bacteria were diluted in RPMI (RPMI; Gibco, Paisley, UK) to a concentration of 1 × 10^6^ CFU/mL. Stock solutions of sphingosine 20 mM in 7.5% n-octyl-β-D-glucopyranoside (OGP) were prepared by sonication for 10 min in a water bath. Sphingosine was added to the bacteria (1–20 μM in 0.0075% OGP) and incubated for 1–24 h at 37 °C. Suspensions were then diluted and plated on LB agar.

### 4.16. Statistical Analysis and Quantification

All data were obtained from independent measurements and expressed as arithmetic mean ± standard deviation (SD). We then tested the results for normal distribution using the David Pearson–Stephens test. Statistical analysis was performed using Student’s *t*-test for single comparisons and ANOVA for multiple comparisons. GraphPad Prism 9.0.0 statistical software (GraphPad Software, La Jolla, CA, USA) was used for the analyses. All data were quantified using ImageJ Version 1.53t and are expressed as arbitrary units (a.u.).

## Data Availability

Not applicable.

## Abbreviations

CF	cystic fibrosis
CFTR	human cystic fibrosis transmembrane conductance regulator
Cftr	mouse cystic fibrosis transmembrane conductance regulator
*M. abscessus* ssp. *abscessus*	*Mycobacterium abscessus* ssp. *abscessus*
